# Japan’s contribution to strengthening global health emergency workforce capacity through the Global Outbreak Alert and Response Network

**DOI:** 10.5365/wpsar.2024.15.5.1302

**Published:** 2025-09-17

**Authors:** Yukimasa Matsuzawa, Kanae Takagi, Haruka Iwasaki, Sangnim Lee, Hidetoshi Nomoto, Masahiro Ishikane, Mugen Ujiie, Sharon Salmon, Norio Ohmagari

**Affiliations:** a Global Outbreak Intelligence, Capacity Building and Deployment Coordination Center, Disease Control and Prevention Center, National Center for Global Health and Medicine, Japan Institute for Health Security, Tokyo, Japan.; b Disease Control and Prevention Center, National Center for Global Health and Medicine, Japan Institute for Health Security, Tokyo, Japan.; c Department of Respiratory Medicine, National Center for Global Health and Medicine, Japan Institute for Health Security, Tokyo, Japan.; d Department of Epidemiology and Clinical Research, The Research Institute of Tuberculosis, Japan Anti-Tuberculosis Association, Tokyo, Japan.; e World Health Organization Regional Office for the Western Pacific, Manila, Philippines.; f UNSW Medicine and Health, School of Population Health, University of New South Wales, Sydney, New South Wales, Australia.

The COVID-19 pandemic served as a stark reminder of how rapidly infectious disease threats can escalate into global crises and of the critical importance of a well prepared, agile and coordinated emergency workforce to contain them. At the heart of this global readiness is the Global Outbreak Alert and Response Network (GOARN), a trusted mechanism established by the World Health Organization (WHO) and powered by a worldwide collaboration of more than 320 partners. GOARN enables the rapid deployment of experts during outbreaks and plays a central role in strengthening the long-term capacity, coordination and resilience needed to address public health emergencies. (*1*)

Building on Japan’s National Action Plan for Strengthening Measures on Emerging Infectious Diseases (*2*) and in alignment with GOARN’s mission (*3*) to coordinate international responses to outbreaks and to support capacities to detect and contain health threats, the Government of Japan launched its Global Health Strategy (*4*) in May 2022; the Strategy signals a renewed commitment to global health security, especially in strengthening prevention and preparedness for, and responses to, public health crises, and promoting collaboration and surge support during international health emergencies. Informed by lessons from the COVID-19 pandemic, this Strategy aims to support the development of a resilient global health architecture through enhanced prevention, preparedness and response activities. Both GOARN and Japan’s Global Health Strategy recognize that global collaboration is essential for building an agile and capable health emergency workforce. This shared vision laid the foundation for Japan’s deeper engagement with GOARN, resulting in increased Network activities and the participation of domestic institutions. This Perspective article outlines Japan’s contributions towards strengthening the global health emergency workforce through GOARN, highlighting efforts to advance coordinated outbreak preparedness, emergency response and long-term capacity-strengthening.

As of May 2025, 19 institutions in Japan are GOARN partners, including hospitals, universities and national authorities (**Box 1**). (*5*) This level of participation reflects Japan’s strong commitment to ensuring global outbreak preparedness and response. To build on this foundation, the Global Outbreak Intelligence, Capacity Building and Deployment Coordination Center (GIC) was established in December 2022 within Japan’s National Center for Global Health and Medicine (NCGM). The GIC has a dual mandate to enhance domestic expertise and to strengthen engagement with global networks. As the focal point for GOARN activities in Japan, the GIC supports outbreak preparedness and response by delivering GOARN trainings, facilitating the use of outbreak intelligence, coordinating deployments of experts during public health emergencies, and working closely with WHO and other international partners. The GIC also provides localized access to GOARN resources through a dedicated Japanese-language web site. (*6*)

Box 1 **GOARN partners in Japan**

1. Association of Medical Doctors of Asia
2. Hokkaido University
3. Hyogo Medical University
4. Japan International Cooperation Agency
5. Japanese Red Cross Wakayama Medical Center
6. Kurume University
7. Mie National University
8. Ministry of Health, Labour and Welfare
9. Nagasaki University
10. Nara Medical University
11. National Center for Global Health and Medicine
12. National Institute of Infectious Diseases
13. Niigata University
14. Osaka Metropolitan University
15. Saint Mary’s Hospital
16. Tohoku University
17. Toshima Hospital
18. University of Osaka
19. University of Tokyo

## Strengthening the emergency workforce through training and capacity-building

In response to lessons identified from the 2014 outbreak of Ebola virus disease in West Africa and the 2015 outbreak of Middle East respiratory syndrome in Republic of Korea, the Government of Japan identified the need to further strengthen both domestic and global response capabilities for threats from infectious diseases. Japan developed a comprehensive action plan to enhance its surveillance, coordination and rapid response capacities as part of its contribution to global health security.

Since 2019, the NCGM, a designated WHO Collaborating Centre for Prevention, Preparedness and Response to Emerging Infectious Diseases, has participated in and delivered GOARN’s multitiered training programme, (*7*) in addition to contributing more broadly to global health security and outbreak responses. In close collaboration with WHO headquarters, the WHO Regional Office for the Western Pacific and other GOARN partners, the NCGM has organized six annual sessions of the Tier 1.5 training, introduction to international outbreak response, for 175 Japanese and other regional public health experts since 2019. This intermediate-level training, bridging Tier 1 and Tier 2, emphasizes a multidisciplinary approach to deployments and team-based coordination across specialties, such as epidemiology, laboratory diagnostics, clinical case management, and infection prevention and control. It also strengthens communication, teamwork and problem-solving skills through interactive workshops, preparing both Japanese and other regional experts for deployments during international outbreaks. (*8*)

The NCGM identified staff to participate in and be trainers for GOARN’s Tier 2 training, which uses an outbreak response scenario, held in New Delhi, India, in 2022. This intensive course is designed to simulate the conditions encountered during deployments for international outbreaks, providing a safe environment for multidisciplinary teams to apply their technical knowledge while adapting to the complex realities of responding to outbreaks in unfamiliar settings. (*9*)

Outbreak response leaders from several Japanese GOARN partner institutions have also participated in the Tier 3 training, designed to address leadership during outbreaks, including in the inaugural training in Berlin, Germany, and the first women-focused leadership course in Darwin, Australia.

To ensure the continuity and sustainability of training efforts, 14 Japanese experts have been trained as trainers, contributing to both domestic and international GOARN capacity-building activities. Japan has also deployed these trainers to support GOARN training workshops organized by partner institutions in other countries.

To further strengthen the engagement of Japanese GOARN partners, the NCGM, with support from the Ministry of Health, Labour and Welfare, convened two meetings of national GOARN focal points in November 2022 and November 2023. These meetings facilitated the exchange of information about deployment experiences, coordination mechanisms and institutional readiness, and reinforced Japan’s commitment to strengthening its role in responses to global outbreaks through GOARN. (*10*)

## Facilitating deployments through a national roster

From 28 January 2020 to 12 May 2025, GOARN partners in Japan submitted 147 offers of assistance, resulting in the deployment of 50 individuals to 16 operations across 15 countries. In collaboration with the Ministry of Health, Labour and Welfare, the NCGM established a streamlined process for mobilizing Japanese experts in response to GOARN requests, using the Japan GOARN roster mailing list. This roster includes more than 200 experts available for rapid deployment. The initiative has also strengthened collaboration among Japanese institutions, thereby enhancing national capacity to contribute to responses to global health emergencies.

As the threat of future pandemics persists, GOARN partners in Japan remain committed to strengthening outbreak response capacity – domestically, regionally and globally – through sustained collaboration, capacity development and rapid deployment mechanisms. By serving as active members of GOARN, Japanese partners bridge global needs with their national expertise, exemplifying how strategic national engagement can enhance global health security. Looking ahead, continued investment in workforce development, cross-border collaboration and systems for rapid responses will be essential to building a safer and more resilient future for global health.
